# Transcultural Adaptation and Validation of the Fonseca Anamnestic Index in a Spanish Population with Temporomandibular Disorders

**DOI:** 10.3390/jcm9103230

**Published:** 2020-10-09

**Authors:** Carmen María Sánchez-Torrelo, Noelia Zagalaz-Anula, Roger Alonso-Royo, Alfonso Javier Ibáñez-Vera, Jesús López Collantes, Daniel Rodríguez-Almagro, Esteban Obrero-Gaitán, Rafael Lomas-Vega

**Affiliations:** 1 FisioMedic Clinic, Dos Hermanas, 41701 Sevilla, Spain; fisiomedic.dh@gmail.com (C.M.S.-T.); rar00032@red.ujaen.es (R.A.-R.); 2 Department of Health Sciences, Campus de las Lagunillas, University of Jaén, 23071 Jaén, Spain; ajibanez@ujaen.es (A.J.I.-V.); dralmagro4@gmail.com (D.R.-A.); eobrero@ujaen.es (E.O.-G.); rlomas@ujaen.es (R.L.-V.); 3 Dental Medical Center Drs. López Collantes, Dos Hermanas, 41701 Sevilla, Spain; citas@lopezcollantes.es

**Keywords:** temporomandibular joint disorders, surveys and questionnaires validation studies, reproducibility of results

## Abstract

Background: The Fonseca Anamnestic Index (FAI) offers a simple method to screen temporomandibular disorders (TMD). This study aimed to validate the Spanish version of the FAI in patients with TMD. Methods: The sample consisted of 125 subjects (66 TMD and 59 controls) aged over 18 years. Construct validity, internal consistency, test-retest reliability, concurrent validity and capacity to discriminate between TMD and healthy subjects were analyzed. Results: The Spanish version of the FAI showed a structure formed by three factors. Cronbach’s alpha was 0.826. The reliability of the items varied between substantial to almost perfect and was excellent for the total score (intraclass correlation coefficient = 0.937). The standard error of measurement (SEM) was 6.52, with a minimum detectable change (MDC) of 12.78. FAI score showed a significant correlation with headache, neck pain and vertigo measurements. A cut-off point >35 showed a sensitivity = 83.33% and a specificity = 77.97% in differentiating between healthy and TMD patients, with an area under the curve (AUC) = 0.865. Conclusions: The Spanish version of the FAI is a valid and reliable instrument for diagnosing people with TMD, with appropriate general clinimetric properties. Discrimination between patients with and without TMD is excellent.

## 1. Introduction

Temporomandibular disorders (TMDs) are defined as a subgroup of craniofacial pain problems that involve the temporomandibular joint (TMJ), masticatory muscles and associated head and neck musculoskeletal structures [1]. TMDs are the most common orofacial pain condition of non-dental origin. Tenderness and pain of the masticatory muscles, pain in the TMJ, limited jaw joint movements, a clicking or crackling sound on the TMJ grinding and wearing of the teeth, headache, associated dizziness, hearing loss and tinnitus are frequent symptoms [2].

The etiology of TMDs is considered multifactorial and is related to parafunctional habits, bruxism, body posture, stress, age, gender, malocclusion, trauma, rheumatic diseases, overload, and other systemic factors such as fibromyalgia, low back pain, spinal pain, chronic fatigue syndrome, irritable bowel syndrome, sleep disorders, tension and migraine headaches and allergies [2,3].

In the general population, the prevalence of TMDs ranges from 5 to 12% [4], and approximately 50% of affected patients suffer from orofacial pain or will experience it in the future [5]. A greater female prevalence has been described in the scientific literature, with a female-to-male ratio of up to 4:1 [4].

The main generally accepted clinical examination of cranial-mandibular joint dysfunctions is based on the diagnostic criteria for temporomandibular disorders (DC/TMD) protocol [6], which includes an extensive and complex battery of tests and questions. The DC/TMD protocol is a validated tool for diagnosing the most common conditions of orofacial pain related to TMDs; it was derived from the original DC/TMD protocol, which was even more extensive and complex than this latest version. It is a test administered by a clinician that consists of 12 items and evaluates muscle and joint pain, measurements of the different movements of opening, closing, right and left lateralization and protrusion made in centimeters, headaches in the last 30 days, type of bite, opening pattern, movements, noises (clicks and crackles), joint blockages, pain on palpation, TMJ and muscle pathologies. However, the DC/TMD protocol is still too complicated and takes a long time to administer, and the examiner needs to have been previously trained.

The Fonseca anamnestic index (FAI) was developed and validated by Dr. Dickson da Fonseca in Sao Paulo, Brazil in 1992 [7,8]. Its structure consists of 10 questions with a three-point scale (0 = no, 5 = sometimes and 10 = yes), with the overall score of the test ranging from 0 to 100. The FAI evaluates the presence or absence of symptoms caused by TMDs and their severity (mild, moderate and severe). Although the DC/TMD protocol is a standardized and widely used test for the diagnosis of TMDs, the complexity of its use has resulted in other, less difficult diagnostic tests, such as the FAI, being used frequently. Additionally, the FAI can be self-completed by the patient. However, despite its ease of use and application in different countries, the FAI has not been validated for use in the Spanish population.

This study aimed to validate the standard version of the FAI in the Spanish population and to analyze the clinimetric properties of the Spanish version of the FAI in patients with TMDs.

## 2. Materials and Methods

### 2.1. Participants

To meet the objectives of this study, a cross-sectional questionnaire validation study was designed. This study received the approval of the Research Ethics Committee of Jaén, Spain (Internal code 1539-N-19. Date of approval 26 September 2019). All participants provided written informed consent to participate in this study, which was conducted in accordance with the Declaration of Helsinki, good clinical practices, and all applicable laws and regulations.

For the calculation of the sample size, the criterion was to recruit a minimum of 10 subjects per item of the questionnaire to be validated [9] with a minimum of 100 patients [10]. The study was developed between October and December 2019. The sample was selected from the patients of the FisioMedic clinic (Dos Hermanas, Sevilla, Spain) who attended the physiotherapy, general medicine and traumatology services and from those of the Dental Medical Center Drs. López Collantes who attended stomatology services (Dos Hermanas, Sevilla, Spain). Recruitment was performed by personal interview after a first telephone contact. In all, 208 people were contacted, but the final sample was composed of 125 participants (66 TMDs patients and 59 healthy controls). Sociodemographic and anthropometric characteristics of the groups are shown in Table 1. 

Patients 18 years or older who were diagnosed with TMDs were eligible for this study. Patients with severe neurological or psychiatric pathology that prevented the correct completion of the questionnaires and measures provided for in the study were excluded. In addition, a sample of healthy controls without pathology of TMDs among those who did not meet the diagnostic criteria for TMDs was selected to test the ability of the FAI to discriminate between patients and controls.

### 2.2. Cross-Cultural Adaptation

For cross-cultural adaptation of the original Portuguese version of the FAI to the Spanish version, the international quality of life assessment project for cross-cultural translation [11] was followed. First, the Portuguese version of the FAI was independently translated into Spanish by two bilingual experts. A single version of the FAI was developed by consensus between translators and researchers. In the next stage, two bilingual experts translated the Spanish version back into Portuguese. The Portuguese-translated contents were then compared by the investigators with the original Portuguese version of the FAI to verify whether they had achieved semantic, linguistic, conceptual, and technical equivalence. Finally, to test its viability, the Spanish version of the questionnaire was completed by 20 participants to verify that they were able to understand the questions, instructions, and answering options. The time required to complete the questionnaire was 3–4 min. The Spanish version of the FAI appears in Supplementary Table S1.

### 2.3. Measurements

Before completing the questionnaires, including the FAI, all the patients were interviewed to collect demographic data such as age, sex, height, weight, BMI, educational level, work situation, smoking habits, alcoholic habits and physical activity.

Compliance with the diagnostic criteria for temporomandibular disorders was verified using the DC/TMD examination protocol [12]. This protocol consists of 12 different sections evaluating muscle and joint pain, metric measurements of jaw movements such as opening, closing, lateralization and protrusion, whether the patient experienced headache in the last 30 days, type of bite, opening pattern, movements, joint noises, joint blockages, and pain on palpation; finally, by means of a diagnostic tree and a scheme, the protocol determined whether the diagnostic criteria according to the findings found reached a diagnostic conclusion. The study used a simplification of the results to differentiate between patients with TMDs and those without it and to be able to use it as the gold standard when compared with the results of FAI.

The numeric pain rating scale (NPRS) is a self-implemented pain intensity perception scale. In this test, all the possibilities are arranged at the same level, with 0 being the absence of pain and 10 being the maximum pain the patient is capable of imagining, organized in an increasing manner from left to right; the patient only has to mark with a cross the answer considered correct [13]. In the present study, the patients recorded orofacial pain and neck pain on two independent NPRS pain scales.

In this study, health status was measured with the 12-item short-form health survey (SF-12). The SF-12 is a simple and quick questionnaire compared to its predecessor, the SF-36 which is self-administered and evaluates general quality of life from physical and emotional points of view. It consists of 12 questions that are presented with a variable number of answers. The final result of the test is obtained in a more exact way by means of a statistical processing instrument that provides the value of the physical and mental summary scores with values between 0 and 100 [14].

Dizziness and vertigo sensations were measured with the dizziness handicap inventory (DHI), which is a self-implemented scale that identifies vertigo or lack of balance. The instrument consists of 25 questions that can be answered as yes, no or sometimes. This questionnaire identifies functional, physical and emotional problems related to balance disorders. Each dimension corresponds to different questions distributed randomly throughout the test. The functionality questions correspond to items 3, 5, 6, 7, 12, 14, 16, 19 and 24, the emotional questions correspond to items 2, 9, 10, 15, 18, 20, 21, 22 and 23, and the questions on the physical dimension correspond to items 1, 4, 8, 11, 13, 17 and 25 [15,16].

The headache impact test (HIT-6) is a self-administered headache questionnaire that consists of six questions with five possible answers. The possible outcomes are “never”, “rarely”, “sometimes”, “very often” and “always”. The numerical result is the sum of the answers. The HIT-6 has been adapted for use in a multitude of languages and cultures, including peninsular Spanish [17].

The neck disability index (NDI) is a questionnaire that assesses disability produced by neck pain. It consists of ten questions with six different answers that are ordered from least to most disability, with 0 corresponding to no disability and 5 corresponding to greatest disability. The result is the sum of the answers, ranging from 0 to 50. The categorization of the final result is as follows: “No disability” if the result is between 0 and 4, “moderate disability” between 15 and 24, and “complete disability” between 35 and 50 [18].

### 2.4. Statistical Analysis

Data management and analysis were performed with the SPSS 20.0 statistical package (SPSS Inc., Chicago, IL, USA) and MedCalc Statistical Software version 19.1.5 (MedCalc Software bv, Ostend, Belgium) [19].Descriptive analysis was performed using means and standard deviations for continuous variables and frequencies and percentages for categorical variables. The Kolmogorov-Smirnov test was used for the analysis of the normality of quantitative variables, and the Levenne test was used to verify the homoscedasticity of the samples. We worked with a 95% confidence level (*p* < 0.05).

The construct validity was evaluated by exploratory factorial analysis (factorial validity) using principal component analysis (PCA) with varimax-type orthogonal rotation. Bartlett’s sphericity test and the Kaiser-Meyer-Olkin test (KMO) [20] were administered.

The Shrout and Fleiss type 2.1 intraclass correlation coefficient (ICC) was used to measure the test-retest reliability of the total test score [21]. Reliability was considered poor when the ICC was <0.40, moderate when the ICC was between 0.40 and 0.75, substantial when the ICC was between 0.75 and 0.90, and excellent when the ICC was >0.90. From this coefficient, the standard error of measurement (SEM) and the minimum detectable change (MDC) were found. The SEM was calculated as the baseline standard deviation (SD) (σbase) minus the square root of (1-Rxx), where Rxx is the test-retest reliability index (ICC) [22]. The agreement between the two observations of each item was analyzed using the Kappa coefficient weighted by quadratic weights [23]. The agreement was considered null if Kappa <0.00, insignificant if Kappa was between 0.00–0.20, discreet if Kappa was between 0.21–0.40, moderate if Kappa was between 0.41–0.60, substantial if kappa was between 0.61–0.80 and almost perfect if Kappa was between 0.81–1.00 [24]. In addition, the MDC was quantified at the 95% confidence level (MDC95) from the SEM formula as follows: MDC95 = 1.96 * σbase * "√ (1-ICC), where 1.96 is the z-value corresponding to the 95% confidence interval (MDC95). The MDC provides a good tool for translating the ICC into units of change in the instrument. In addition, Bland-Altman charts were generated to evaluate the limits of agreement [25].

Internal consistency was measured using Cronbach’s alpha coefficient. The alpha coefficient is considered poor if it was less than 0.70, and good if it was between 0.70 and 0.90; when it was greater than 0.90, it is interpreted as indicating the existence of redundancy [26].

To analyze the concurrent validity of the FAI with the NDI, DHI, HIT-6, SF-12 and NPRS, Pearson’s correlation coefficient r was used. The correlation coefficient is considered strong if it is >0.50 and moderate if it was between 0.30 and 0.50 [27].

The ability to discriminate between patients and controls was performed using receiver operating characteristic (ROC) curves. Initially, patients with or without TMDs were classified based on the diagnostic criteria of the DC/TMD protocol, and the score obtained in the FAI was evaluated as the variable. In the ROC curve, the fraction of true positives (sensitivity) was represented as a function of the fraction of false positives for different cut-off points. The area under the curve (AUC) was also calculated as a measure of the parameters abilities to discriminate between the two diagnostic groups (subjects with or without TMDs). The AUC was considered statistically significant when the 95% confidence interval did not include 0.5 [28]. Values between 0.5 and 0.7 indicated low accuracy, values between 0.7 and 0.9 indicated good accuracy, and values greater than 0.9 indicated high accuracy [29].

## 3. Results

One hundred twenty-five patients met the eligibility criteria and completed the planned evaluations. Of these, 66 presented with TMDs, and 59 were healthy controls. There were statistical significant differences between the two samples of TMD and Healthy subjects in gender, height, weight and academic level (Table 1).

**Figure 1:**
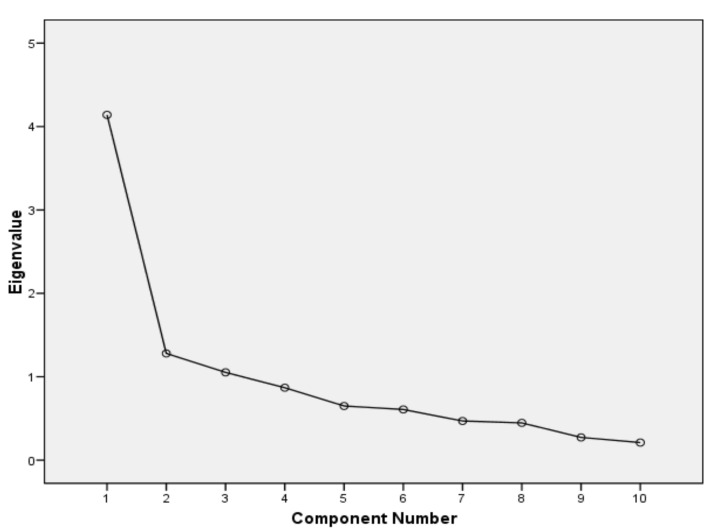
Scree plot for factorial analysis.

Construct validity measured by factor analysis showed a structure with three factors (Figure 1), the first of which included items 1, 2, 3, 6, 7 and 8, the second factor included items 4 and 5, while the third factor included items 9 and 10 (Table 2). This three-factor structure explained 64% of the variance (Table 3). The measure KMO = 0.802 (*p* < 0.001), indicating that the sample could be considered appropriate for factor analysis. In Figure 1 and Table 3 it can be seen that there are three factors that present eigenvalues greater than 1, which is usually the criterion to retain them. Between these three factors they retain more than 60% of the variance of the data, which is usually the minimum criterion in social and health sciences.

The internal consistency analysis showed a Cronbach’s alpha = 0.826, indicating good internal consistency. Analysis of the items (Table 4) showed that the elimination of item 10 resulted in a slight improvement in Cronbach’s alpha, although in general, all items seem to contribute adequately to the consistency of the test, with decreases in the alpha value observed when each item is deleted.

**Figure 2:**
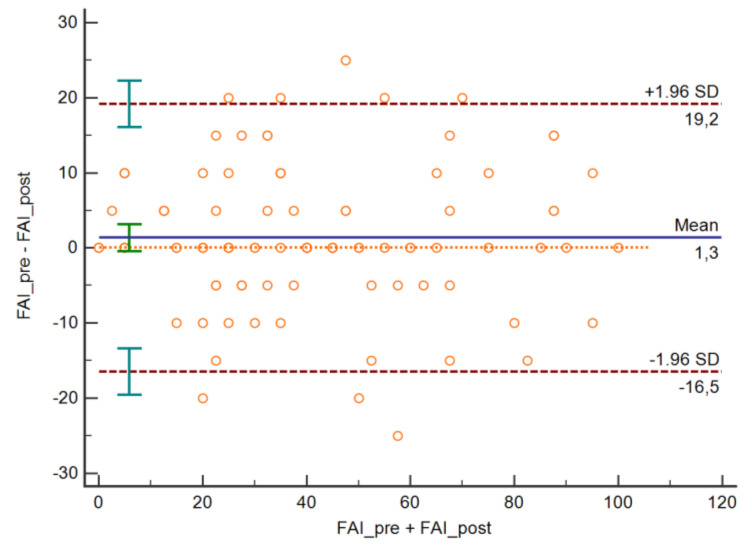
Bland-Altman plot. FAI: Fonseca Anamnestic Index.

The test-retest reliability analysis (Table 5) showed weighted Kappa values between a minimum of 0.654 in item 1 and a maximum of 0.898 in item 4, indicating a reliability that varied between substantial and almost perfect. The ICC value for the overall scale score was excellent. The SEM was 6.52 points, and the MDC was 12.78 points. The Bland-Altman plot is shown in Figure 2.

In the concurrent validity analysis, the Spanish version of the FAI showed significant correlation with the other indices of TMDs assessment as well as with measures of headache and neck pain and the evaluation of vertigo. However, the correlation with the SF-12 PCS was not statistically significant (Table 6). In general, the correlation with the SF-12 components was poor, moderate with the measures of headache and vertigo, and strong with the orofacial NPRS score and with measures of neck pain.

**Figure 3:**
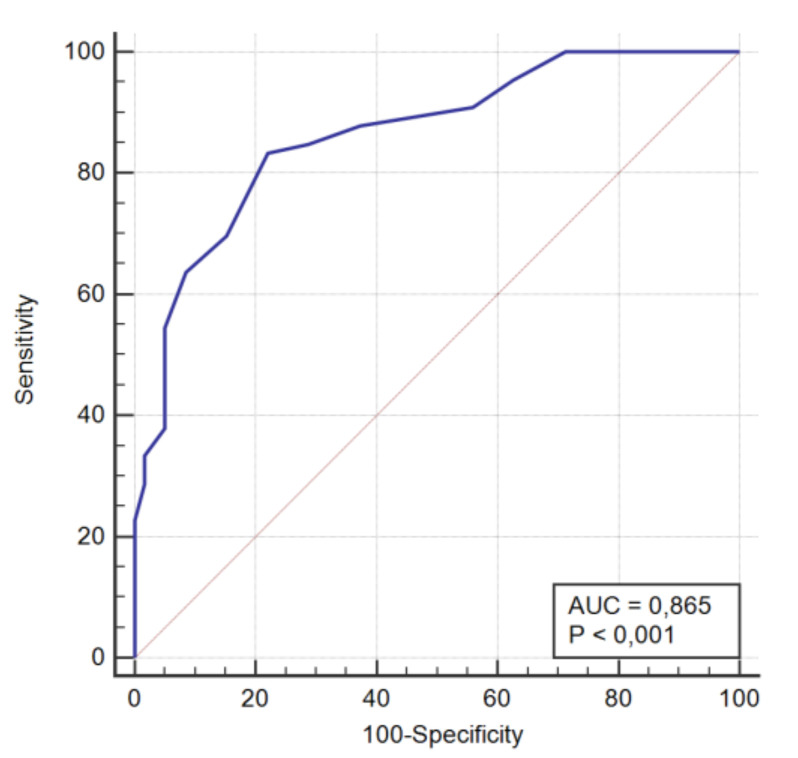
ROC curve of the FAI for discriminating between patients and controls. AUC: Area Under the Curve.

In the ROC curve analysis, the ability of the Spanish version of the FAI to discriminate between patients with TMDs and healthy subjects was evaluated with the AUC, which had a mean of 0.865 (0.792 to 0.919; *p* < 0.001) (Figure 3). With a cut-off point of > 35 points, the FAI showed a sensitivity of 83.33%, corresponding to the proportion of TMDs patients detected, and a specificity of 77.97%, corresponding to the proportion of healthy individuals detected. The remaining predictive values are shown in Table 7.

## 4. Discussion

The present study evaluated the clinimetric properties of the Spanish version of the FAI, which has been suggested to be a valid and reliable instrument for assessing patients with TMDs and the degree of severity of the condition and for discriminating between patients with or without TMDs. A total of 66 patients with TMDs and 59 controls self-administered the test, and the time spent to complete it was approximately 3–4 min. The two groups were comparable except for a higher proportion of women in the sample of TMD patients. This caused secondary differences such as lower weight and height in the TMD group, as well as a higher proportion of subjects with university studies, due to the higher proportion of university graduates among the Spanish female population [30]. Once the Chinese version of the questionnaire has been obtained for use in the largest linguistic community, obtaining the version in Spanish can serve as the basis for the extension of this questionnaire in the second largest linguistic community in the world.

To the best of our knowledge, this is the most complete clinimetric study of any version of the FAI. The FAI test-retest reliability had been previously analyzed for the Chinese version in a study from Zhang et al. [31], but only in terms of the total score, which showed an ICC = 0.823, which is less than the excellent value of the ICC (0.937) that was found in the Spanish version. Additionally, we studied the reliability of each item using the nonparametric statistic corresponding to the ICC, the weighted Kappa. The different items showed a reliability between substantial and almost perfect. From the ICC value, we also calculate the SEM and the MDC. To the best of our knowledge, this contribution from our study is absolutely original.

Another original contribution of our study is the measurement of concurrent validity with measures of quality of life, pain and factors related to TMDs. In the Chinese version, Zhang et al. [31] performed a very original calculation using an FAI cut-off value > 15 points to determine the agreement with the diagnosis from the DC/TMD axis, arriving at a good Kappa value (0.633).

In our study, we examined the construct validity by exploratory factorial analysis, resulting in a FAI structure compatible with a multidimensional, three-factor structure. The first factor was composed of items 1, 2, 3, 6, 7 and 8. The second factor was formed by 4 and 5 items, while items 9 and 10 corresponded with the third component. Our results are similar to those obtained by Rodrigues-Bigaton et al. [8] by exploratory factorial analysis. In their study, the first factor comprised items 1, 2, 3, 6 and 7, the second items 4, 5 and 10 and the third items 8 and 9. This structure differed from that obtained by Campos et al. [32] via confirmatory factorial analysis.

In our study, we also measured internal consistency using Cronbach’s alpha. Our results showed good internal consistency (Cronbach’s alpha = 0.826). This result is better than that reported by Campos et al. (Cronbach’s alpha = 0.745) [32], which can also be classified as good and, in any case, indicates that there was no redundancy between the items. However, the Chinese version obtained poor internal consistency (Cronbach’s alpha = 0.669) [31].

The accuracy of the FAI in identifying myogenic TMDs had been previously analyzed by Berni et al. 2015 [33], who obtained high accuracy by taking a cut-off point > 45 points in the FAI (AUC = 0.940). In this study, the RDC/TMD protocol was taken as the gold standard. In our case, with the same methodology, we obtained good accuracy when a cut-off point > 35 points was taken in the FAI (AUC = 0.865). In our study were obtained values of sensitivity and specificity of 83.33% and 77.97%, respectively. However, the validation of the Chinese version shows a higher ability to detect true positives (sensitivity of 95.9%) but a poorer ability to differentiate true negatives (specificity = 71.9%) [31] than the Spanish version.

Some limitations of the present study should be considered. First, as in all previous studies, it includes a very high proportion of female patients due to the higher prevalence of TMDs in the female population. Second, although the sample was sufficient for the respective analyses, the number of participants in our study was lower than in other reference studies. Moreover, there are several psychometric properties that can be analyzed in the instrument. Although our study analyzed the most common psychometric properties, others remain to be studied, such as the sensitivity to change or the ability to discriminate between different types of populations.

## 5. Conclusions

The findings of this study confirm that the Spanish version of the FAI has good internal consistency, test-retest reliability, and construct and concurrent validity. Moreover, the Spanish version of the FAI has shown very satisfactory general psychometric properties and is able to discriminate between patients with and without TMDs.

## Figures and Tables

**Table 1:** Sociodemographic characteristics of the sample.

VARIABLES		NO TMD (*n* = 59)		TMD (*n* = 66)		*p*-Value
**Continuous**		**Mean**	**SD**	**Mean**	**SD**	
Weight (Kg)		77.64	18.92	68.73	14.50	0.004
Height (m)		1.65	0.09	1.61	0.08	0.001
Body Mass Index		28.44	7.04	26.79	6.74	0.183
Age (Years)		47	15	43	13	0.113
**Categorical**		**F**	%	**F**	%	
Gender	Female	35	59.3%	61	92.4%	
	Male	24	40.7%	5	7.6%	<0.001
Physical Activity	No	20	33.9%	29	43.9%	
	Yes	39	66.1%	37	56.1%	0.253
Worker Out Home	No	14	23.7%	13	19.7%	
	Yes	45	76.3%	53	80.3%	0.586
Economic Level	<20.000	37	62.7%	38	57.6%	
	>20.000	22	37.3%	28	42.4%	0.560
Academic Level	Primary	15	25.4%	7	10.6%	
	Secondary	31	52.5%	30	45.5%	
	University	13	22.0%	29	43.9%	0.013
Smoke Habit	No	36	61.0%	46	69.7%	
	Smoker	8	13.6%	7	10.6%	
	Ocasional Smoker	6	10.2%	6	9.1%	
	Exsmoker	9	15.3%	7	10.6%	0.766
Alcoholic Habit	No	22	37.3%	21	31.8%	
	Drinker	4	6.8%	2	3.0%	
	Ocasional Drinker	33	55.9%	43	65.2%	0.445

TMD: temporomandibular disorders; SD: standard deviation; F: frequency.

**Table 2:** Rotated component matrix of the Fonseca anamnestic index (FAI) factor analysis.

	Component		
	1 ^a^	2 ^a^	3 ^a^
1. Do you have difficulty opening your mouth wide?	0.843		
2. Do you have difficulty moving your jaw to the sides?	0.836		
3. Do you feel fatigue or muscle pain when you chew?	0.816		
4. Do you have frequent headaches?		0.842	
5. Do you have neck pain or stiff neck?		0.825	
6. Do you have earaches or pain in temporomandibular joint?	0.703		
7. Have you ever noticed any noise in your temporomandibular joint while chewing or opening your mouth?	0.521		
8. Do you have any habits such as clenching or grinding your teeth?	0.617		
9. Do you feel that your teeth do not come together well?			0.746
10. Do you consider yourself a tense (nervous) person?			0.699

^a^ Factors obtained from FAI factor analysis.

**Table 3:** Percentages of variance explained by the factor analysis performed using principal component analysis.

Component	Initial Eigenvalues			Extraction Sums of Squared Loadings			Rotation Sums Of Squared Loadings		
	Total	% of Variance ^a^	Cumulative % ^b^	Total	% of Variance ^a^	Cumulative % ^b^	Total	% of Variance ^a^	Cumulative % ^b^
1	4.140	41.402	41.402	4.140	41.402	41.402	3.376	33.758	33.758
2	1.280	12.800	54.203	1.280	12.800	54.203	1.557	15.570	49.328
3	1.053	10.528	64.731	1.053	10.528	64.731	1.540	15.403	64.731
4	0.869	8.686	73.417						
5	0.650	6.498	79.915						
6	0.608	6.080	85.995						
7	0.470	4.699	90.694						
8	0.447	4.469	95.163						
9	0.273	2.729	97.892						
10	0.211	2.108	100.000						

^a^ Percentage of variance that explains each factor of the questionnaire structure. ^b^ Total percentage of variance explained jointly by the factors that compose the questionnaire structure.

**Table 4:** Item analysis of the Spanish version of the Fonseca anamnestic index.

	Mean of the Scale if the Element Is Deleted	Scale Variance if the Element Is Removed	Corrected Total-Element Correlation	Multiple Squared Correlation	Alfa De Cronbach if Element Is Deleted ^a^
**ITEM 1**	19.09	22.258	0.600	0.573	0.803
**ITEM 2**	19.07	21.890	0.639	0.641	0.799
**ITEM 3**	19.37	20.202	0.748	0.671	0.784
**ITEM 4**	19.66	22.647	0.402	0.316	0.821
**ITEM 5**	19.94	22.818	0.425	0.329	0.818
**ITEM 6**	19.46	21.686	0.532	0.417	0.808
**ITEM 7**	19.44	21.668	0.544	0.381	0.806
**ITEM 8**	19.70	20.294	0.624	0.504	0.797
**ITEM 9**	19.46	21.863	0.445	0.333	0.818
**ITEM10**	19.94	24.360	0.211	0.099	0.838

^a^ Cronbach’s alpha value if the item is deleted from the analysis. Item 1–10: Questions of the Fonseca anamnestic index.

**Table 5:** Reliability of the items and Fonseca anamnestic index total score.

ITEM	Weighted Kappa	Lower Bound	Upper Bound	Reliability
**ITEM 1**	0.654	0.460	0.847	Substantial
**ITEM 2**	0.773	0.630	0.916	Substantial
**ITEM 3**	0.801	0.694	0.907	Almost Perfect
**ITEM 4**	0.898	0.850	0.947	Almost Perfect
**ITEM 5**	0.684	0.524	0.844	Substantial
**ITEM 6**	0.764	0.663	0.865	Substantial
**ITEM 7**	0.703	0.571	0.835	Substantial
**ITEM 8**	0.860	0.773	0.947	Almost Perfect
**ITEM 9**	0.854	0.762	0.945	Almost Perfect
**ITEM 10**	0.694	0.557	0.831	Substantial
**TOTAL SCORE ^a^**	0.937	0.908	0.957	Excellent

^a^ Intraclass correlation coefficient (ICC) value for the overall Fonseca anamnestic index score. Item 1–10: Questions of the Fonseca anamnestic index.

**Table 6:** Concurrent validity measured by Pearson Correlation.

VARIABLE	r Coefficient	*p*-Value	Correlation
HIT-6	0.387	<0.001	Moderate
NDI	0.512	<0.001	Strong
SF-12 PCS	−0.063	0.491	Poor
SF-12 MCS	−0.184	0.041	Poor
NPRS Cervical	0.507	<0.001	Strong
NPRS Orofacial	0.731	<0.001	Strong
DHI Functional	0.442	<0.001	Moderate
DHI Emotional	0.419	<0.001	Moderate
DHI Physical	0.418	<0.001	Moderate

HIT-6: the headache impact test; NDI: neck disability index; SF-12 PCS: short-form health survey physical component summary; SF-12 MCS: short-form health survey mental component summary; NPRS: numeric pain rating scale; DHI: dizziness handicap inventory.

**Table 7:** Predictive values of Fonseca anamnestic index to diagnostic case of temporomandibular disorder.

Criterion	Sensitivity	95% CI	Specificity	95% CI	+LR	95% CI	-LR	95% CI	+PV	95% CI	-PV	95% CI
>35	83.33	72.1– 91.4	77.97	65.3–87.7	3.78	2.3–6.2	0.21	0.1–0.4	80.9	72.1–87.4	80.7	70.6–87.9

95% CI: 95% confidence interval; +LR: positive likelihood ratio; -LR: negative likelihood ratio; +PV: positive predictive value; -PV: negative predictive value.

## Supplementary Materials

The following are available online at https://www.mdpi.com/2077-0383/9/10/3230/s1, Table S1: Índice Anamnésico de Fonseca. Versión Española
