# Supplementary material for: Transcultural Adaptation and Validation of the Fonseca Anamnestic Index in a Spanish Population with Temporomandibular Disorders
Source: J Clin Med. 2020 Oct 9;9(10):3230. doi: 10.3390/jcm9103230 (PMC7600423; doi:10.3390/jcm9103230)
Supplement: Supplementary file 1 [file jcm-09-03230-s001.pdf]

Índice Anamnésico de Fonseca. Versión Española.

Por favor, responda a las 10 preguntas siguientes con NO, A VECES o SI. Marque solo una respuesta para cada pregunta.

	**NO**	**A VECES**	**SÍ**
¿Tiene dificultad para abrir la boca?			
¿Tiene dificultad para mover la mandíbula de lado a lado?			
¿Siente fatiga o dolor muscular al masticar?			
¿Tiene dolores de cabeza frecuentes?			
¿Tiene dolor de cuello o tortícolis?			
¿Tiene dolor de oído o dolor en sus articulaciones temporomandibulares?			
¿Ha notado algún clic en su articulación temporomandibular al masticar o al abrir la boca?			
¿Ha notado si tiene la costumbre de apretar o rechinar los dientes?			
¿Siente que sus dientes no encajan bien?			
¿Se considera una persona tensa (nerviosa)?			
